# Supplementary material for: In vivo interaction screening reveals liver-derived constraints to metastasis
Source: Nature. 2024 Jul 24;632(8024):411–8. doi: 10.1038/s41586-024-07715-3 (PMC11306111; doi:10.1038/s41586-024-07715-3)

nature portfolio

https://doi.org/10.1038/s41586-024-07715-3

Supplementary information

In vivo interaction screening reveals liver-derived constraints to metastasis

In the format provided by the authors and unedited

Supplemental Gating Strategy

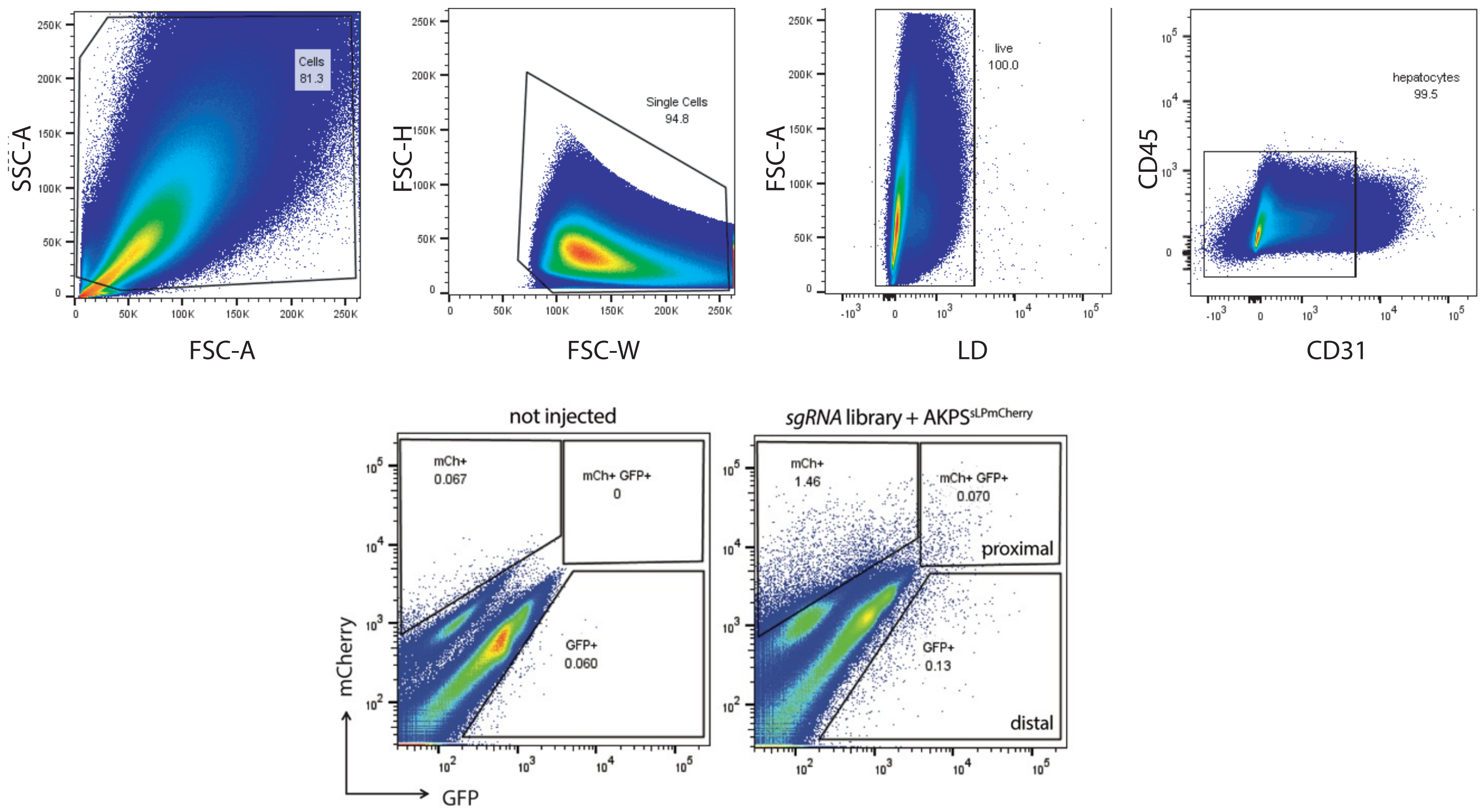

Supplement: Supplementary file 1 — The gating strategy used for sorting of GFP+mCherry+ hepatocytes. [file 41586_2024_7715_MOESM1_ESM.pdf]
