# Supplementary material for: In vivo interaction screening reveals liver-derived constraints to metastasis
Source: Nature. 2024 Jul 24;632(8024):411–8. doi: 10.1038/s41586-024-07715-3 (PMC11306111; doi:10.1038/s41586-024-07715-3)
Supplement: Supplementary file 2 — Reporting Summary [file 41586_2024_7715_MOESM2_ESM.pdf]

nature portfolio

Corresponding author(s): Andreas E. Moor

Last updated by author(s): 17/5/24

Reporting Summary

Nature Portfolio wishes to improve the reproducibility of the work that we publish. This form provides structure for consistency and transparency in reporting. For further information on Nature Portfolio policies, see our Editorial Policies and the Editorial Policy Checklist.

Statistics

For all statistical analyses, confirm that the following items are present in the figure legend, table legend, main text, or Methods section.

n/a	Confirmed	
☐	☒	The exact sample size (*n*) for each experimental group/condition, given as a discrete number and unit of measurement
☐	☒	A statement on whether measurements were taken from distinct samples or whether the same sample was measured repeatedly
☐	☒	The statistical test(s) used AND whether they are one- or two-sided<br>*Only common tests should be described solely by name; describe more complex techniques in the Methods section.*
☐	☒	A description of all covariates tested
☐	☒	A description of any assumptions or corrections, such as tests of normality and adjustment for multiple comparisons
☐	☒	A full description of the statistical parameters including central tendency (e.g. means) or other basic estimates (e.g. regression coefficient) AND variation (e.g. standard deviation) or associated estimates of uncertainty (e.g. confidence intervals)
☐	☒	For null hypothesis testing, the test statistic (e.g. *F*, *t*, *r*) with confidence intervals, effect sizes, degrees of freedom and *P* value noted<br>*Give P values as exact values whenever suitable.*
☒	☐	For Bayesian analysis, information on the choice of priors and Markov chain Monte Carlo settings
☒	☐	For hierarchical and complex designs, identification of the appropriate level for tests and full reporting of outcomes
☐	☒	Estimates of effect sizes (e.g. Cohen's *d*, Pearson's *r*), indicating how they were calculated

Our web collection on statistics for biologists contains articles on many of the points above.

Software and code

Policy information about availability of computer code

Data collection	The code used in this study is available at: https://github.com/Moors-Code/coco_mosaic_liver
Data analysis	GraphPad Prism v8.2.0 GraphPad Software Schneider https://www.graphpad.com/scientific- software/prism/<br>R software 4.1.0 GNU project https://www.r-project.org<br>R Studio Server 1.4.1717, RStudio https://www.rstudio.com<br>Bcl2fastq v2.20.0.422 (Illumina https://support.illumina.com/sequencing/sequencing_software/bcl2fastq-conversion-software.html)<br>Space Ranger (v1.1.0 or v1.2.0, 10x Genomics)<br>edgeR R package Robinson et al, 2010 https://bioconductor.org/packages/release/bioc/html/edgeR.html<br>msigdbr R package R Bioconductor https://cran.r-project.org/web/packages/msigdbr/vignettes/msigdbr-intro.html<br>fgsea R package Sergushichev et al, 2016 https://bioconductor.org/packages/release/bioc/html/fgsea.html<br>ggplot2 R package Wickham, 2016 https://cloud.r-project.org/web/packages/ggplot2/index.html<br>NicheNet v1.0.0 R package, Browaeys et al, 2019<br>CellPhoneDB v2.0.0 R package, Efremova et al, 2020<br>Seurat v4, R package, Hao, Hao et al, 2021<br>Signac v1.12, R package, Stuart et al, 2021<br>DecontX v1.0.0, R package, Yang et al, 2020<br>Biomart R package v2.46.3<br>Image J Fiji Schindelin et al, 2012 https://imagej.net/Fiji/<br>FlowJo v10.7.1 (Becton Dickinson & Company)<br>cutadapt, Martin et al 2011<br>FISHquant v2, python package, Imbert et al, 2022

HORIZON v2.1.0.0, Lunaphore technologies
Bowtie2, Langmead et al, 2012
MAgECK, Li et al, 2014

For manuscripts utilizing custom algorithms or software that are central to the research but not yet described in published literature, software must be made available to editors and reviewers. We strongly encourage code deposition in a community repository (e.g. GitHub). See the Nature Portfolio guidelines for submitting code & software for further information.

Data

Policy information about availability of data

All manuscripts must include a data availability statement. This statement should provide the following information, where applicable:

- Accession codes, unique identifiers, or web links for publicly available datasets
- A description of any restrictions on data availability
- For clinical datasets or third party data, please ensure that the statement adheres to our policy

The sequencing data generated in this study are available at the Gene Expression Omnibus under the accession numbers GSE267981 and GSE267982 and the Zenodo repository: 10.5281/zenodo.7737590. The code used in this study is available at https://github.com/Moors-Code/coco_mosaic_liver.
Kaplan-Meier analysis of 1211 CRC patients was performed using the online tool http://kmplot.com. Recurrence free survival (RFS) was stratified by SEMA4A, SEMA4C, SEMA4D and SEMA4G expression in the Affymetrix colon dataset, using best cutoff.
Protein atlas stainings: SEMA4A, D, C, and G antibody stainings were obtained from the Human Protein Atlas with the R package HPAanalyze.
CNV analysis of class IV semaphorin genes: the CNV status of SEMA4A, D, C, and G in 290 CRC patients was obtained from the TGCA-COAD dataset88.
Published scRNASeq datasets: Pre-processed and annotated scRNASeq profiles of epithelial cells in the KUL and Samsung dataset were obtained from the Synapse repository syn34942428. scRNASeq datasets of matched liver metastases and primary tumors were obtained from the Gene Expression Omnibus under the accession numbers GSE22585786 and GSE17831887, and imported in Seurat. Epithelial cells were subsetted based on EPCAM expression. Expression of was computed with the AddModuleScore function.

Research involving human participants, their data, or biological material

Policy information about studies with human participants or human data. See also policy information about sex, gender (identity/presentation), and sexual orientation and race, ethnicity and racism.

Reporting on sex and gender	na
Reporting on race, ethnicity, or other socially relevant groupings	na
Population characteristics	The sample of human CRC hepatic metastasis (#CB522586) originated from a 44 years old male.
Recruitment	na
Ethics oversight	CRC-patient derived organoids from primary and liver metastases were obtained from the University Hospital Basel following patient consent and ethical approval (Ethics Committee of Basel, EKBB, number EKBB numbers 2019-00816).

Note that full information on the approval of the study protocol must also be provided in the manuscript.

Field-specific reporting

Please select the one below that is the best fit for your research. If you are not sure, read the appropriate sections before making your selection.

☒ Life sciences ☐ Behavioural & social sciences ☐ Ecological, evolutionary & environmental sciences

For a reference copy of the document with all sections, see nature.com/documents/nr-reporting-summary-flat.pdf

Life sciences study design

All studies must disclose on these points even when the disclosure is negative.

Sample size	In accordance with the 3Rs, the smallest sample size was chosen that could give a significant difference. Given the robustness of the phenotypes across all methods used, the minimum sample size assuming no overlap in control versus experimental is three animals per experiment.
Data exclusions	No animals were excluded, unless data acquisition quality was insufficient. For experiments with adeno-associated virus, animals with GFP coverage < 60% were excluded. Thresholds applied in bioinformatic analyses (standard parameters) are available in the github repository of the code.
Replication	Data was combined from independent biological replicates (mice) and analyzed together. Only consistent observations were reported. When indicated in the figure legends, data from multiple experiments was pooled for quantification.

Randomization	Treatment and control animals were randomized across cages, sex and age. For other experiments, randomization was not required.
Blinding	The researcher was blinded to the genotype or treatment group during the processing and analysis.

Reporting for specific materials, systems and methods

We require information from authors about some types of materials, experimental systems and methods used in many studies. Here, indicate whether each material, system or method listed is relevant to your study. If you are not sure if a list item applies to your research, read the appropriate section before selecting a response.

Materials & experimental systems

n/a	Involved in the study
☐	☒ Antibodies
☐	☒ Eukaryotic cell lines
☒	☐ Palaeontology and archaeology
☐	☒ Animals and other organisms
☒	☐ Clinical data
☒	☐ Dual use research of concern
☒	☐ Plants

Methods

n/a	Involved in the study
☒	☐ ChIP-seq
☐	☒ Flow cytometry
☒	☐ MRI-based neuroimaging

Antibodies

Antibodies used

For sorting, hepatocytes were resuspended in 2 mL FACS buffer (2 mM EDTA, 0.5% BSA in PBS) and stained with Zombie Violet (1:500) (Biolegend #423113), TruStain FcX™ (anti-mouse CD16/32) antibody (BioLegend #101320, 1:50), PE/Cy7 anti-mouse CD31 (BioLegend #102418, 1:300), BV570 anti-mouse CD45 (BioLegend #103135, 1:300) for 25 min at 4 °C.

Immunofluorescence on formalin fixed paraffin embedded tissue: Sections were incubated overnight at 4°C with the following primary antibodies (1:100, in blocking buffer): anti-CD146 (Abcam #ab75769); anti-α-SMA (Abcam #ab5694) anti-periostin (Abcam #ab227049) and anti-GFP (Aves Labs #GFP-1020). Sections were repeatedly washed in PBST and incubated with the following secondary antibodies (1:400, in blocking buffer) for 1h at RT: AlexaFluor goat anti-rabbit 594 (#A-11012), AlexaFluor goat anti-rabbit 647 (#A-21244), AlexaFluor goat anti-chicken 647(#A32933), all from ThermoFisher.

Multiplexed immunofluorescence was performed on the Comet instrument (Lunaphore) with the following antibodies (1:100): anti-cleaved Caspase 3 (Cell Signaling #9661), anti-CD68 (Abcam #ab125212), anti-CD4 (Abcam #ab183685), anti-Ki67 (Abcam #ab15580), anti-E-cadherin (Cell Signaling, #3195), anti-α-SMA (1:1000, SIGMA #A2547), anti-CD146 (Abcam, #ab75769).

Immunofluorescence on fixed frozen tissue: Sections were stained as above with the following primary antibodies: anti-glutamine synthetase (1:100, Biolegend #856201), anti c-Myc (1:100, 9E10, Thermo Fisher Scientific), anti-Plexin B2-PE (1:500, Biolegend #145903), anti-GFP-AlexaFluor488 (1:200, ThermoFisher #A-21311), anti-Zeb1 (1:400, Novus #NBP1-05987), anti-α-SMA (1:1000, SIGMA #A2547), anti-E-cadherin (1:100, Biotechne, AF748), anti-Epcam (1:100, Abcam #2884975), anti-GRHL2 (1:100, Abcam #ab271023), anti-Klf4 (1:100, Biotechne #AF3158), anti-ELF3 (1:100, Thermo Fisher Sceintific #PA5-120996) and anti-Sema4A (1:100, Biolegend #148402). DAPI counterstain, mounting and imaging was performed as above. F-actin was stained by incubating blocked slides for 2 hours at RT with Alexa Fluor 647 Phalloidin (1:400, Invitrogen #A22287). Sections were repeatedly washed in PBST and incubated with the following secondary antibodies (1:400, in blocking buffer) for 1h at RT: AlexaFluor goat anti-rabbit 594 (#A-11012), AlexaFluor goat anti-rabbit 647 (#A-21244), AlexaFluor goat anti-chicken 647(#A32933), all from ThermoFisher.

Immunofluorescence in organoids: anti-Zeb1 (1:400, Novus #NBP1-05987), anti-Ecadherin (1:200, BD Biosciences #610181), mouse anti-Sema4A (Biolegend #148402), rat anti-Sema4C-AF647 (Biotechne #FAB8497R), rat anti-Sema4D-PE (Biolegend #147603), rabbit anti-Sema4G (Thermo Fisher Scientific #BS-11479R).

Immunofluorescence in cell lines: rabbit anti-VSV-G antibody (Thermo Fisher Scientific #PA1-29903), mouse anti-PlexinB2-PE (Biolegend #145903).

Validation

All antibodies have been previously validated by the manufacturer for use in immunofluorescence. See manufacturer's website for further information. All antibodies were additionally validated in the study by including an unstained control (no primary antibody, only secondary).

Eukaryotic cell lines

Policy information about cell lines and Sex and Gender in Research

Cell line source(s)

HEK293T cells - ATCC
AML12 - CRL-2254 - ATCC
PTA-5565 H2B-mCherry (Mohamed Bentires-Alj, University of Basel)

	KPC cell line (Ilaria Guccini, ETH Zurich)<br>D4M-3A (Merck Millipore)
Authentication	None of the cell lines were autheticated
Mycoplasma contamination	Cells tested negative for micoplasma contamination.
Commonly misidentified lines<br>(See ICLAC register)	No commonly misidentified cell lines were used in this study.

Animals and other research organisms

Policy information about studies involving animals; ARRIVE guidelines recommended for reporting animal research, and Sex and Gender in Research

Laboratory animals	Albumin-Cre mice (AlbCre, stock no. 003574), LSL-dCas9-SPH (dCas9-SPH, stock no. 031645), LSL-Cas9 (Cas9, stock no. 024858), mT/mG mice (stock no. 007576) and Plxnb2flox/flox mice (stock no. 036883) were obtained from a local live mouse repository. Chow and water were available ad libitum, unless specified. All mice were in the B6J background and maintained on a 12h light / 12h darkness schedule. Mice were housed and bred under specific pathogen-free conditions in accredited animal facilities. All experiments were performed on 6-16 week-old male and female mice.
Wild animals	No wild animals were used.
Reporting on sex	All experiments were performed on 6-16 week-old male and female mice.
Field-collected samples	No field-collected samples.
Ethics oversight	All experimental procedures were performed in accordance with Swiss Federal regulations and approved by the Cantonal Veterinary Office.

Note that full information on the approval of the study protocol must also be provided in the manuscript.

Flow Cytometry

Plots

Confirm that:

☒ The axis labels state the marker and fluorochrome used (e.g. CD4-FITC).

☒ The axis scales are clearly visible. Include numbers along axes only for bottom left plot of group (a 'group' is an analysis of identical markers).

☒ All plots are contour plots with outliers or pseudocolor plots.

☒ A numerical value for number of cells or percentage (with statistics) is provided.

Methodology

Sample preparation	mice were sacrificed by raising CO2 concentrations, then the abdomen was opened and a G22 cannula was inserted into the inferior vena cava. The liver was perfused with 20 mL Hanks buffer (0.5 mM EDTA and 25 mM HEPES in HBSS) followed by 15 mL digestion buffer (15 mM HEPES and 32 µg/mL Liberase in low glucose DMEM). After initial swelling of the liver, the portal vein was cut to allow outflow. After perfusion, the gallbladder was removed and the liver was transferred to a petri dish with 10 mL digestion buffer and squished with a cell scraper to release the hepatocytes. Liberase was inactivated by adding 10 mL isolation buffer (10% fetal bovine serum (FBS) in low glucose DMEM). The cell suspension was filtered through a 100 µm cell strainer and centrifuged at 50 g for 2 min. The supernatant was removed and the pellet was washed again twice with 20 mL isolation buffer. Hepatocytes were resuspended in 2 mL FACS buffer (2 mM EDTA, 0.5% BSA in PBS) and stained with Zombie Violet (1:500) (Biolegend #423113), TruStain FcX™ (anti-mouse CD16/32) antibody (BioLegend #101320, 1:50), PE/Cy7 anti-mouse CD31 (BioLegend #102418, 1:300), BV570 anti-mouse CD45 (BioLegend #103135, 1:300) for 25 min at 4 °C. Hepatocytes were washed and filtered through a 70 µm strainer. CD45-CD31- hepatocytes which contained a sgRNA (GFP+) were divided into metastasis-proximal (mCherry+) and metastasis-distal (mCherry-) by drawing different sorting gates on an AriaIII sorter (BD Biosciences) with 70-micron nozzle. Cells were collected in PBS, spun down at 800 g for 5 min and the pellet was stored at -20 °C.
Instrument	AriaIII sorter (BD Biosciences) with 70-micron nozzle.
Software	Aquired data were analyzed using FlowJo software.
Cell population abundance	Post-sort populations were not analyzed. Genomic DNA was extracted from all sorted cells.
Gating strategy	Events were initially gated by FSC-A and SSC-A, then by FSC-A and FSC-H (to exclude doublets). Hepatocytes were gated as CD45-CD31-.

☒ Tick this box to confirm that a figure exemplifying the gating strategy is provided in the Supplementary Information.
